# Evolution of storytelling pedagogy in global health course at a U.S. Native American-Serving Nontribal Institution from Fall 2019 to Spring 2023

**DOI:** 10.3389/fpubh.2023.1165241

**Published:** 2023-09-29

**Authors:** Tapati Dutta, Camille Keith

**Affiliations:** Fort Lewis College, Durango, CO, United States

**Keywords:** storytelling, global health course, pedagogy, COVID-19, Native American-Serving Nontribal Institution (NASNTI)

## Abstract

**Background and purpose:**

Responding to COVID-19-induced disruptions to traditional teaching methodologies, and considering the relevance of narratives among indigenous populations, “storytelling as pedagogy” was developed and implemented in the undergraduate Global Health course in a Native American-Serving Nontribal Institution (NASNTI) in Colorado.

**Methods:**

We describe the evolving pedagogic adjustments and storytelling strategies incorporated into the global health course from Fall 2019 to Spring 2023. This entailed before the COVID-19 in-person format, online digital storytelling during the pandemic emergency, the HyFlex and hybrid classes with the emergence of “new normals,” and finally the gradual move to in-person classes. The story arc in the course included the following: (1) Course learning outcomes revisited and the course syllabus language framed based on the native philosophies of empowerment education and experiential learning, (2) students’ inputs sought to incorporate socioculturally responsive topics in the course syllabus (e.g., dental health disparities among indigenous populations), (3) strategic and non-threatening shifts such as “no textbooks” and “no finals” introduced, (4) global health thought partners invited by the course instructor and coached to use story-based teaching methods, (5) use of first-person trauma-informed storytelling methods to teach specific global health topics, and (6) students undertook gratitude journaling, a scaffolding exercise of writing letters on global health topics to global health thought partners.

**Results:**

Storytelling as pedagogy was most effective in the in-person format, while digital storytelling during the COVID-19-induced online classes was extremely challenging considering the stark digital divide in the Navajo Nation. First-person, trauma-informed storytelling is a helpful approach to discuss insider–outsider perspectives and can potentially establish sustainable trustworthy relationships among the students, instructor, and global health thought leaders. Gratitude journaling and photovoice can be tweaked as powerful storytelling methods to build students’ interaction-based critical thinking, intercultural humility, and professional networking.

**Conclusion:**

Mapping storytelling pedagogies’ best practices can be useful in developing a granulated understanding of this strategy and utilizing them across diverse disciplines in higher education. Faculty capacity building is recommended to enable the former to conceptualize culturally responsive storytelling pedagogies and create assessment plans to assess students’ learning outcomes through the utilization of this method.

## Introduction

The power of storytelling as a pedagogic strategy is increasingly being acknowledged by general, scientific, and technical courses because of their deep appeal, ability to validate experiences, transcend cultural, mythical, personal, and sacred knowledge, and enable students to construct their life narratives (1, 2). Furthermore, storytelling in public health and allied disciplines facilitates exploring, reflecting on the realities of clinical practices, and developing empathy (3, 4). There are several examples of successful usage of storytelling as an effective active learning and high-impact pedagogical strategy. For example, a three-part storytelling approach assisted students to construct life narratives in which they were able to internalize, evolve, and integrate their stories with each other (5), or an instance of English as Foreign Language classrooms in Indonesia used narrative texts that facilitated students become aware of the moral lessons of the story, aroused students’ imagination, and increased student’s literacy interest (6). Existing scholarship also showcases this method being used as a research and intervention tool to examine health risks and experiences, understand and influence public opinion on prevention issues, inform public health practice, and engage populations, clinical professionals, and organizations (7).

That said, there could be four key issues that prevent storytelling from being the learning paradigm, especially in majority–minority institutions of higher education in the United States. First, active learning and storytelling methods need to be planned and implemented in an inclusive way, especially among ethnic minorities and first-generation students (8). Second, narrative models of teaching might reflect a tendency toward a transactional approach—an action-orientated comprehensive approach of teaching and learning with elements of mutuality, reciprocity, and giving back to the community—and thus could be “distanced” from the students and generate superficial sensitivities when infrequently used as a tool (9). Third, there are rare instances with minority-serving academic institutions where teaching–learning is co-developed as an interactional process—for example, designing the course or teaching some of the issues raised by students or periodically undertaking ‘how is this class going’ with students —rather than as an instructional deliberation that can subsequently fail to link students’ agency to racial, feminist, or minority tensions (10). In addition, there is a dearth of professional folklorists (in the US sense of the term) practicing storytelling pedagogy, which invariably results in very uneven quality accounts of this methodology (11).

This study summarizes how storytelling was devised as pedagogy and practiced as an integral strategy in the global health course in a Native American-Serving Nontribal Institution (NASNTI) during the changing COVID-19 scenarios, from before the coronavirus disease 2019 (COVID-19) period (Fall 2019), during the first 2 years of the pandemic (Spring 2020–Fall 2021), and with the emergence of “new normals.” (Spring–Spring 2023). We further advocate that story-based teaching methods can potentially develop a strong feeling of connection and sense of belonging among the students, course instructor, external thought partners, and, most importantly, the community, who are protagonists in several case stories that are discussed in this course.

## Learning environment

This NASNTI is in southwestern Colorado and noted by the 2021 US News & World Report as the ninth most diverse liberal arts college in the nation, defined by its rural and multiethnic composition (12). The college offers 59 undergraduate majors and enrolls approximately 3,000 undergraduate and 200 graduate students annually. With representation from 17 countries, 48 states in the United States, and 185 Native American tribes and Alaska Native villages, the college has 58% students of color and 46% first-generation students (13).

The global health course taught in this college is an upper level (PH300-level) undergraduate Public Health course capped at 25 students. It is one of the public health major electives, and since Fall 2019 has been taught every semester by one of the authors (TD). Most students who take this course are majoring in disciplines such as Public Health, Health Sciences, and Psychology, while others are from backgrounds such as Business, Management, Engineering, Native American Studies, Sociology, and Biochemistry. Students in the class are mostly Native American, first-generation graduate aspirants, and especially during the pandemic showcased deep interest and commitment in studying the topic and investing their acumen to improve the health of vulnerable communities (14, 15).

## Pedagogical philosophies

The unique mix of indigeneity and intersectionalities of NASNTI including a range of sociocultural diversities makes it crucial to acknowledge the relevance of broader narrative contexts when developing and teaching public health courses in this college. Furthermore, the complex history of the college as an “American Indian” boarding school resonated with the narratives of health disparities, untold stories of uncounted numbers, and power asymmetries in global health (16). The combination of the residents and history of the college, the philosophies of narrative medicine and oral traditions practiced among indigenous communities (17, 18), plus the pandemic exposing the need for a more sensitive and empathetic future public health workforce illustrated the possibilities of using storytelling pedagogy in the global health course. Additionally, the course instructor’s personal and professional experience with vulnerable communities (19), training in health education (20), participatory planning, monitoring evaluation, and managing for impact (21), and digital storytelling from StoryCenter® (22) were deep influencers to her praxis-oriented teaching that are incumbent to agentic skills such as respectful listening, empathy, and classroom community building.

## Pedagogical format: building the story arc

In addition to the course instructor’s own background of experiential learning, her knack for storytelling processes, and foreseeing storytelling as an effective pedagogic strategy in this course, as a new employee to the college in Fall 2019, she leaned heavily on the previous existing course materials to develop the course modules. In doing this, she observed that the previous materials highlighted the binaries of haves versus have-nots, developed versus developing, communicable and non-communicable. These materials also reflected a stark lack of ongoing social, institutional, and political overlaps such as intersectionalities, acknowledging that everyone has their own unique experiences of discrimination, which is a complex interplay and function of identity factors such as race, gender, class, disability, and sexuality; glo-cal that juxtaposes ‘global’ and ‘local’ and emphasizes a constant influence of globalization on the local and, on the other hand highlights local, nuanced reinterpretation that gets a voice on the global diaspora (23) and relative deprivation in health discussing dimensions of health inequalities and inequities by geography, race, ethnicity, gender, education, class, income, and occupation (24). Teaching from this biomedical perspective was often evident in the reductionist and pessimistic framing, such as the “developing” countries and communities thereof being mentioned as third-world countries and uneducated communities.

Simultaneously, the course instructor reviewed the Course Learning Outcomes (CLOs) of the college that are guided by the Council on Education for Public Health (CEPH) guidelines (page 26), which has an impetus on curriculum that focuses on “the socioeconomic, behavioral, biological, environmental, and other factors that impact human health and contribute to health disparities” (25). Keeping the CLOs in mind, the course instructor started conceptualizing story-based teaching methods that resonated advancing the college’s “graduates ready to promote and advance the health of communities.” By this means, student-centric skills of critical thinking, professionalism, information literacy in issues pertaining to global health, and intercultural humility, “a lifelong process of self-reflection and self-critique whereby the individual not only learns about another culture but also one starts with an examination of her/his own beliefs, cultural identities, and explicit and implicit biases,” were also underpinned (26).

Thereafter, the instructor applied for and received a faculty development Learning Circle grant (US$500) in Spring 2020 that proposed to collaborate with two other faculties in the college (MC and DG) and use story-based techniques in this course. However, because of the COVID-19 pandemic at the beginning of Spring 2020 and subsequent school closure, the course was rushed to a completely online format. Adding to the turmoil of the pandemic was the formidable digital divide in this region; the in-person planned activities through the grant could not be carried out. The grant was rather utilized in exploring and revising pandemic suited culturally responsive teaching pedagogies, brainstorming with the Teaching Learning Services department to devise a NASNTI tailored definition of hybrid, HyFlex and online, and making all efforts during those almost incommunicado times to identify potential guest speakers who would be interested to share their high-impact stories of practice and research online with students.

Following this, in the latter part of Spring 2020 and continuing through 2021 as COVID-19 disruptions and shutdowns continued, the college adopted diverse teaching methods. They were (i) in-person classes with a hybrid element (synchronous classes that used hybrid elements, such as meeting in outdoor spaces, namely, in tents on certain days and virtually on other days of the week), (ii) completely online classes (asynchronous, taught 100% virtually), (iii) remote access classes, and (iv) HyFlex (hybrid and flexible) classes where the course was “delivered with fully remote option(s)—synchronous or asynchronous—along with regularly scheduled face-to-face classes, allowing students to transition seamlessly between the two learning environments.” (27). At that time, the global health course was delivered in the HyFlex format by utilizing the new e-learning infrastructure that was installed in the college and technology bundles that were made available to students for free or at affordable prices. HyFlex formats allowed capitalizing on technology and offered students greater flexibility to choose the learning modality that suited them best. Because the pandemic amplified academic inequalities that, in turn, affected the psychological wellbeing of both the students and teachers, narratives, vignettes, case stories, and short audiovisuals were incorporated into the course with the expectation to make the classes more interesting and interactive. This marked the budding phases of the story arc. In the HyFlex format, global health classes were delivered synchronously to in-person students and via Zoom (Zoom Inc.). Additionally, each Zoom session and chat were recorded and shared with students who would access the class materials asynchronously.

COVID-19-induced turmoil continued through the Spring 2022 and Fall 2022 semesters. This resulted in the extension of HyFlex teaching of the global health course with nimble transitioning to in-person formats during Spring 2023. This phase was the hallmark of initiating students’ involvement and seeking their input to inform and enrich the course syllabus.

The next step noted the gradual incorporation of interaction between students, academics, and community members. In this case, the course instructor first utilized her professional network and purposefully selected 12 “global health thought partners.” Most of these thought partners are from minority backgrounds themselves or have served minority communities, trained in medicine, public health, psychology, community health ethics, and vaccine trust science, and have illustrative experience of more than 15 years, typified by multi-country program leadership and grant management. All were involved in evidence-based policymaking and influencing roles. Thus, these individuals, by virtue of their knowledge, experience, and positions, were able to provide a unique big-picture perspective of a global health topic. Evites were sent to all 12 thought partners, requesting them to contribute to this class, of whom 8 responded in affirmation (Table 1). The eight thought partners were then inducted by the instructor about the NASNTI and coached to use first-person, trauma-informed storytelling to co-teach sessions elucidating history, evolution, and turning points in global health. Both the thought partners and the instructor were mindful in using trauma-informed sensitivities to teach topics because they were occurring in real time and impacted the Native populations disproportionately (i.e., vaccination equity and gender-based violence) (28).

**Table 1:** Global health thought partners by their qualification specialization and their global health leadership focus areas.

Global health thought partners	Qualification specialization	Global health leadership focus areas
1.	Public Health Administration	Playing a strategic leadership and advocacy role in the Public Health Service and Implementation Science/Division and Office of Tribal Affairs and Strategic Alliances in a federal public health agency.
2.	Medicine, Public Health	Prolific global health contribution in coordinating India’s exemplar polio elimination and leading a donor organization’s global polio vaccine research, product development initiatives and polio-related policymaking across multiple countries and geographies.
3.	Social work, Public Policy	Global strategy formulation, landscapes analysis, and project and partnership management in an international non-profit agency entrusted with addressing micronutrient deficiencies in developing countries.
4.	Dentistry	A dental surgeon with expertise in general dentistry services as well as dental anesthesia in the 4-Corners area, and deeply passionate about pediatric dental health and dental health issues and disparities among NA AI populations.
5.	Medicine, Public health, Health Promotion	Global health expertise in the National Smallpox Eradication Programme in India, the Global Programme on AIDS in Switzerland, and international HIV/AIDS prevention and control, child survival, and reproductive health programs, plus scholarly contribution in the arena of sexual health and sport.
6.	Medicine, Clinical Psychology	Globally acclaimed research practitioner on multi-faceted behavioral and social science research programs focusing on attitudes about and determinants of vaccination with a particular focus on human papillomavirus (HPV) vaccination. Also, one of the core developers of the Multidimensional Scale of Perceived Social Support (MSPSS).
7.	Epidemiology, Public Health	An epidemiologist at a federal public health agency focusing on behaviors associated with the transmission of sexually transmitted infections (STIs), sexual health promotion, social justice, health equity, and dismantling institutional racism.
8.	Medicine, Public Administration	Leading and managing non-profit organizations’ health-related projects internationally, in the times of coups, military rule, and ethnic conflict.

**Figure 1:**
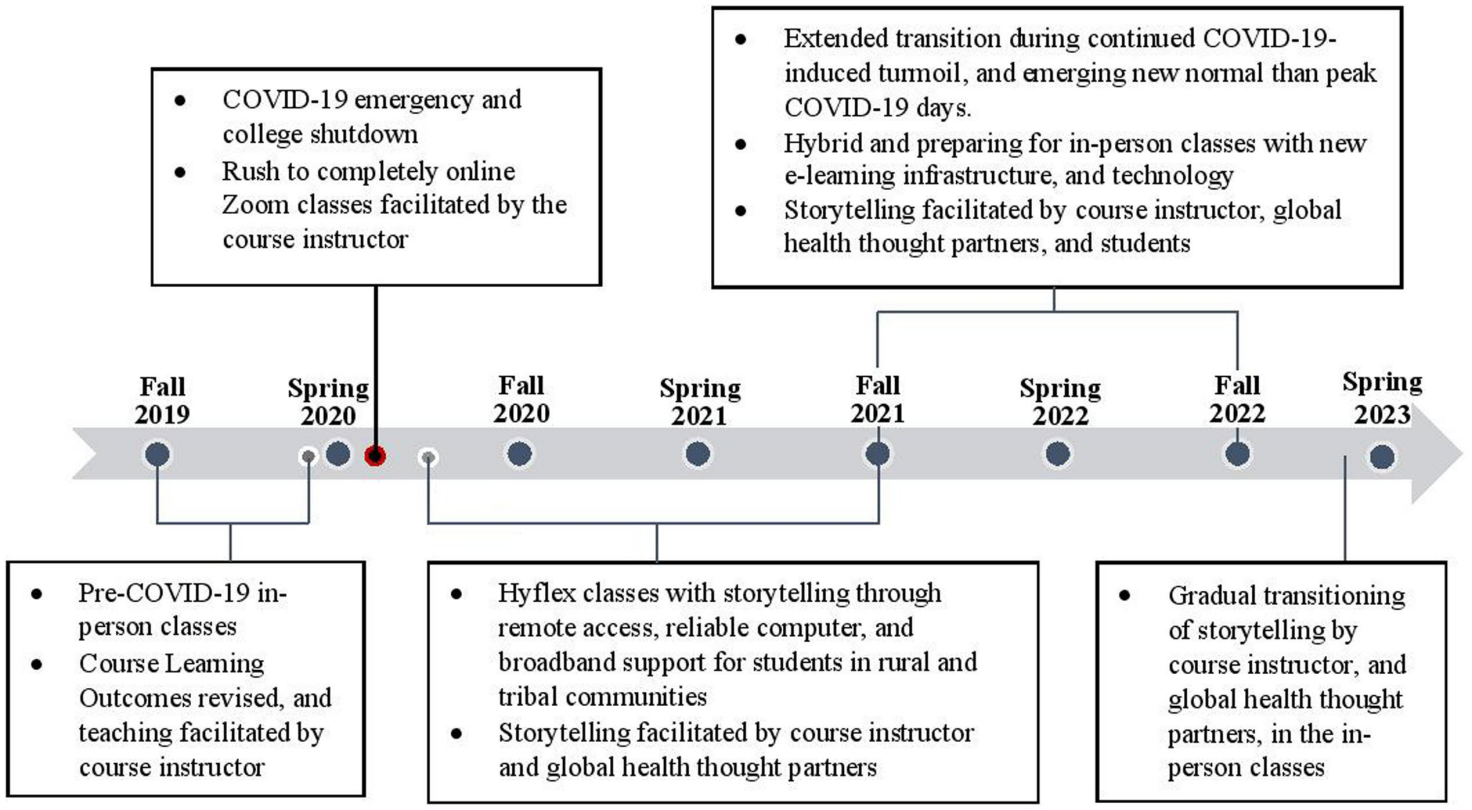
Evolution of storytelling as pedagogy in the global health course in accordance with the college’s COVID-19 response phases, from Fall 2019 to Spring 2023.

The final expression of the story arc transpired as students engaged in more interactive and contemplative activities including think-pair-share groups, role plays, simulations, and reflective gratitude journaling to the global health thought leaders. Figure 1 presents the evolution of storytelling methodologies into the global health course across all eight semesters from Fall 2019 to Spring 2023.

## Results

Overall, the storytelling approach resonated with the diverse traditional storytelling practices and cultures among students in a NASNTI. In addition, the non-threatening story-delivery mood in a classroom was set through the application-based language of the revised Course Learning Outcomes (CLOs) and strategic shifts such as “no textbooks” and “no finals” approaches. The earlier and revised descriptive language of the CLOs is presented in Table 2.

**Table 2:** Comparison of the earlier (prior to Fall 2019) and revised Global Health Course Learning Outcomes (CLOs) (from Spring 2020).

CLOs prior to Fall 2019	Redesigned CLOs from Spring 2020
1. Define and describe the key principles and goals of global health 2. Compare and contrast the traditional biomedical approach with a biopsychosocial approach to global health 3. Compare and contrast how health system structures vary globally 4. Identify key determinants and describe disparities in health outcomes 5. Describe and assess the current global health agenda and prospects for the future	1. Develop an empathy-based epistemological understanding of select global health issues by region and subpopulations 2. Invigorate critical understanding of the evolving field of global health politics: before and during the coronavirus disease 2019 period 3. Enhance knowledgeability of global health stakeholders and improve learning the complex linkages between global and community health 4. Build intercultural humility while identifying key social and political determinants of health and describing health disparities 5. Appreciate implementation science and initiate professional networking with global health stakeholders that will enhance students’ confidence and preparedness to enter the professional workforce

The course instructor’s teaching philosophy as an inclusion strategist, in turn, led to the agentic engagement of students. Instances of this ranged from students’ recommendations and inclusion of topics, such as Community-Based Participatory Research, health disparities among indigenous populations in the United States, dental health among indigenous people in the Four Corners Region, and global health volunteering, to the course.

Attention was given to small things. For example, using the term “thought partners” manifested a sense of collegiality as opposed to the hegemonic term “global health leaders” or “guest speakers” that would convey being external to the classroom community. Storytelling pedagogies included the diverse conceptualizations of health and the varied construct of “global” in global health. This required that everyone involved in the classroom community (instructor, students, and global health thought partners who served as co-teachers in the class) were aware of the emic-etic (insider-outsider) positionalities of each other. Table 3 describes the emic-etic positionalities by classroom stakeholders. Narrative storytelling was effectively used to teach uncomfortable topics, initiate difficult conversations, and enhance student’s critical thinking while exciting them in a way that made room for self-discovery and intercultural humility.

**Table 3:** Classroom community stakeholders’ respective insider and outsider positionalities.

Classroom community stakeholder	Emic (Insider) positionality	Etic (Outsider) positionality
Course instructor	Her global experience influenced her being an inclusion strategist in public health. Her own indigeneity and traditional culture of using narrative storytelling.	Being a first-generation graduate, person of color, and having an international background initially took her more time to build the classroom community.
Students	The resonance of the storytelling with the students’ traditional culture of narrative storytelling methods and (most) students’ exposure and experience of witnessing health disparities helped them make a connection with the latter seamlessly.	Being from small towns and villages often posed as a barrier to visualize themselves as stakeholders in the big picture of global health.
Global health thought partners	Their illustrative journey and experience influenced their passion on global health issues and perspectives thereof that were shared in the class.	Most of them were accustomed to making high-level presentations using traditional methods. For most thought partners it was one of the first experiences to “coteach” in a majority-minority undergraduate class. It took time for some of them to understand the “contemplative spaces and silences” that several students preferred.

The first-person storytelling tools and props that were used by the course instructor and thought partners included narrations of eyewitness accounts, professional journeys, field and lived experiences, excerpts from speeches, and trigger photographs from the field. Stories ranged from folk to scientific domains which made them more relatable as opposed to reading information from a text. These pedagogic processes facilitated linking the past events and the current scenarios (e.g., how experiences from previous pandemics, such as Spanish flu, informed during COVID-19 times) to instill foundational concepts of health policies, disparities, and communication that are not commonly discoursed and critiqued through traditional pedagogy. Trauma-informed storytelling helped to destigmatize public and institutional narratives about poverty and contemplate a holistic definition of health that considered “spiritual dimensions” [of health] beyond the popular definitions that encompass physical, mental, and social wellbeing (29).

Similarly, the usage of audio visuals and still photographs was enlightening in many ways. For example, viewing the documentary series “*The Most Dangerous Ways to School”* (citing example of Bolivia, (30)) helped to elicit shared experiences of risk, resilience, and relative deprivations with the affected communities. Viewing trigger pictures such as seeing ‘happy’ photographs of vaccinators with their family members was a strong tool to deconstruct emotions and experiences while listening to the dreadful stories of the vaccinators being subjected to community backlash and brutally murdered by anti-vaxxers (31).

Stories also entailed exemplification of the thought partners’ and course instructor’s collaborative research that, in turn, encouraged students to consider similar internships/projects in future (32). For example, one thought partner is a clinical psychologist and professor from a public research university who described the development of his *Multidimensional Scale of Perceived Social Support* and acknowledged the course instructor’s translation of the scale into an Indian language. This motivated a few students to undertake language translations of COVID-19 vaccination FAQs retrieved from the Centers for Disease Control and Prevention (CDC) and World Health Organization (WHO) websites into Braille, podcast, and Navajo language–*Diné bizaad* (33). Moreover, one of the students presented this project as the first author at the 50th Annual Symposium on the American Indian organized by the Northeastern State University (34).

In addition to including sessions co-facilitated by global health thought partners in the course, interactive and immersive student activities included role plays, simulations, photovoice, and reflective writings. Box 1 describes an exemplary global health course syllabus in 2022, highlighting each module, the corresponding story-based method that was used to teach each module and the diverse student activities that were undertaken aiming to build classroom community. Two story-based assignments, namely, gratitude journaling and photovoice, are explained here.

(1) Gratitude journaling was a think-pair-share scaffolding exercise undertaken by students in a span of 3–4 weeks. In this exercise, students first chose a peer, formed a pair, and consultatively drafted a letter jointly addressing any one of the global health thought leaders who presented in their class. A typical letter to the thought partner included the following 10 sections: (1) The students thanked the thought partner and gave a brief introduction about themselves to her/him, (2) used five appreciative action words to summarize her/his presentation, (3) described the global health topic that the speaker highlighted and the teaching methods that were used, (4) summarized some of the key aspects that students were grateful for and learned from and related to the presentation (the gratitude condition), (5) cited the Sustainable Development Goals connected to the topic presented, (6) recommended policy-level issues to address the scenario pertaining to this global health issue, (7) suggested programmatic interventions to improve the global scenario (these are very similar to the “hassle condition as defined in gratitude journaling”), (8) opined why this topic is relevant and needed to be learned by global/public health students, (9) described strategic skills that public health students need to address this issue, and (10) suggested ideas for professional networking with the thought leader or their organization in imagining a future research/internship with the thought partner or her/his organization or allied organization. The journaling process was progressive. It required students to express their content knowledge, deliberate structural and systematic factors linked to a global health issue, and ideate professional networking steps with the global health thought leader who had presented on that issue. One such example was students in this class who participated and interacted with the CDC personnel at the 2022 American Indian, Alaska Native, and Native Hawaiian (AIANNH) Career Expo that was organized by the CDC. Accountability was established as each student needed to confirm their respective roles in the completion of this exercise. Gratitude journaling that took shape through almost a month of collaborative working between peers helped (1) students to express their gratitude to the thought partner *via* letters, (2) provided a unique opportunity to thank and acknowledge their peers in the letters, thus expressing one’s own gratitude and enhancing classroom community relationships, and (3) illustrated the complex interplay of emic (insider) and etic (outsider) perspectives of the instructor, thought partners, and themselves. A preliminary version of this method was presented at the 73rd Annual Conference Society for Public Health Education, and components of this study also appeared in the associated abstract for that conference (35).

The potential of this methodology in deepening students’ content understanding, invigorating their empathy, and rekindling our hopes in future graduate ready professionals was evident in excerpts from the gratitude journaling.

Excerpt 1 Responding to the presentation on Impact on health and wellbeing during coups, military rule, and ethnic conflict: The Context of Myanmar, “*Your presentation highlighted the organized, large scaled, and sustained civil war in Myanmar. By becoming emotionally vulnerable, you allowed us as a class, a brief glimpse into health disparities in Myanmar. Students need to be more creative, illustrate adaptive qualities while being present in a humanitarian crisis. There are many forces working to control the people of Myanmar and they will not like the idea of outside help… Students should also be patient, as this issue will likely not get turned around in one or two years.”*

Excerpt 2 Responding to the presentation on Dental health among indigenous populations, “…*Another thing we learned about was the lack of dental knowledge as well as the lack of dentists on Indian reservations. One of the statistics that really stood out was American Indian/Alaska Native had a 400% higher chance of an early childhood dental caries than the general US population… Public health students could learn about these issues and then provide advocacy for these issues through partnership with local governments as well as local dentists on the reservations*.”

Excerpt 3 Responding to the presentation on the overview of COVID-19 health disparities in developing countries. “*Your presentation taught us about COVID-19 challenges in each country … such as income inequality, gender inequality, and inequality of opportunities in India, political challenges in Brazil and gender-based violence in South Africa. Learning more about an issue helps students to figure out what they can do to improve it …and be grateful for all the things that are provided such as clean water, consistent food, health care, quality education, and an overall well-structured life.”*

Excerpt 4 Responding to the presentation on STIs in the USA, “*We appreciate your and TD’s [course instructor’s] commitment to sharing knowledge and being a role model. Growing up on a tribal reservation, STI’s or STD’s are not talked about and are considered a ‘taboo’ topic because of the culture. Fortunately, there has been a rise in conversations and awareness about syphilis among the Navajo people. You are an inspiration to students who have big dreams of working towards reducing health inequities for all. We ask you to continue to advocate and provide opportunities that will open doors (e.g., speaking to Global Health students). Thank you & ahéhee*.”

(2) In the photovoice-based synchronous exercise, students participated in the art exhibition of the college titled “Resilience” and reflected on the exhibited pieces. The experience was unique and offered something new for everyone. It was complex for the course instructor to manage this hybrid exercise with most students participating in-person and a few remote learners *via* Zoom. A professor at the Art & Design department debriefed the students on the art pieces and acknowledged that this was her first time explaining art to non-art students. This exercise allowed interdepartmental synergistic collaboration and showcased three dimensions of learning empowerment as follows: (1) development of new knowledge relating to the pedagogic utility of photovoice as a storytelling approach, otherwise and mostly used as a qualitative research method, (2) stories of resilience embedded in the exhibited pictures instilled respectful perception of vulnerable and marginalized communities, and (3) students were able to build new networks with professors beyond the comfort zones of their academic disciplines. Components of this were published in the college’s magazine, such as FLC voices (36).

BOX 1 **An example of the global health course syllabus in Fall 2022 and story-based teaching methods and student activities used.**

**Table:** 

Global Health Course Modules, Fall 2022	Storytelling methods used	Student activities for agentic engagement and building classroom communities
*Module 1* Millennium Development Goals and Sustainable Development Goals	- World Health Organization documentaries, and interpretation of SDG scores by country	Presurvey of students to understand their contexts better and design student-centered classes and increase students’ relatability to an applied course.
*Module 2* Social Determinants of Health (SDoH) in a Socio-Ecological Framework and key global health organizations	- Case studies, vignettes, and infographics	Students participate in a ‘Digital Storytelling in Health’ webinar by StoryCenter**®** and described unique conceptual learnings, emotional realizations, technological approaches imbibed and how they plan to utilize these learnings in their future public health work.
*Module 3* Violation of ethical principles, formation of international instruments dealing with public health ethics, human rights, and informed consent	- Timeline-based case studies	Students and instructor used Hypothesis® e-learning tool to read, socially annotate and have conversations on the paper ‘Politics of disease control in Africa and the critical role of global health diplomacy A systematic review’.
*Module 4* Health Disparities among indigenous populations	- Emic perspectives of indigenous health policymaker explaining Native American health policies and programs - Emic-etic narratives of a dental surgeon working in the reservation area describing dental health disparities among indigenous populations	Students watched and had discursive comments on TEDx talk *‘Life Lessons via Cannibals, Sex Workers & Marginalized People’*, to improve emotional intelligence, and build a sense of connectedness and empathy with the instructor and glo-cal communities.
*Module 5* The global health endemics-Cancer, HIV and Polio	- Ethnographic journey and mapping comparing HIV and cervical cancer prevention, control, and treatment in developed and developing country - Field stories, and trigger photos explaining the polio outbreak control to eradication	Students answered infographic-based reflection and critical thinking questions.
*Module 6* Implementation science, global health volunteering and community engagement	- Success stories, and collective advocacy in the context of abortion rights - Addressing malnutrition in developing countries through examples of food fortification policies and programs	Students’ mid-course assessment addressing aspects of relevance, relatedness, and reciprocity. Students watched the movie *‘The song of sparrows’* to identify visual moments when ‘boundaries of comforts were pushed’ to watch global health/ public health disparities.
*Module 7* Behavior Change Communication and social media misinformation and disinformation in global health	- Use Photovoice® to discuss on ‘endemics and resilience’	Each student had a choice of watching one spectacular documentary from the series- *‘The Most dangerous Ways to School’*, simulated interning with that community and writing a letter to [the student’s] grandma/ parents, mindfully depicting the issues of health disparity in that place and stressing on the haves, rather than regretting the downsides, and utilizing ‘Appreciative Inquiry’ to describe a hypothetical internship plan.
*Module 8* Emerging issues in Global Health	- Health emergencies explained using narratives of global health personnel in the military and government healthcare	Student pair-up and undertook gratitude journaling to one global health thought partner highlighting: the key aspects that they learned on the topic, the topic’s relevance in public health, strategic public health skills required to address this issue, and recommendations to improve this scenario.

## Discussion

This study reports the evolution of storytelling methods in a NASNTI and is not an evaluation of the said methods in the global health course. Numerous disruptions in 2020, the most critical being the protracted COVID-19 pandemic, and the murder of George Floyd, that led to a paradigm shift in global health education (37, 38). All these called for using ‘stories’ to teach global health methodologies that have been known to disrupt class-, color-, race-, and gender-based stereotypes, diminish stigma, improve addiction-related practices, and reduce fatalistic (e.g., suicidal) ideas (39–42). Using storytelling as a teaching method especially resonated with the student-centric, high-impact experiential, and interdisciplinary learning of the college, invigorated through academia-non-profit and regional partnerships and equity-driven teaching methodologies (43). In addition, storytelling as pedagogy gave students the space to question the dominance of medical frames of reference and discuss the topics of global health as social processes (such as vaccination equity) and aspects that are not readily discussed and critiqued through traditional pedagogy (44).

Reflexivity (intersecting relationships between the course instructor and students) and positionality (ontological and epistemological understanding of issues) were pertinent aspects in integrating storytelling pedagogy into this course. Scholarships on the experience of storytelling as pedagogy explain that such aspects ensure rigor and creditability and deepen understanding of the issues and scientific validity (45). Reflexivity and positionality were also vital because the course instructor, students, and thought partners had diverse ethnic and pedagogic backgrounds and thus held differing orientations about storytelling. Therefore, developing a shared understanding was an evolving process. The global health course was presumably used as the main research and implementation space and thus as cultural insiders or outsiders, that is, emic and etic, necessitating the course instructor’s attention to and exploration of the local cultures while connecting the students with global health themes (46).

The pandemic happening in real time required being sensitive in teaching topics such as social determinants of health because they affected each one of us directly and the Native populations more disparately (47, 48). Using Arundhati Roy’s concept of Pandemic is a Portal (49), storytelling method facilitated to viscerally reveal how identities and socioeconomic status intersected and led to differential outcomes of COVID-19 across individuals, regions, and communities.

In summary, the philosophies of “storytelling pedagogy” can provide with knowledge about intrapersonal, interpersonal, and institutional dimensions that influence everyday classroom practice. This resonated with other research studies reiterating that a well-designed praxis-oriented course can maximize student’s empathetic and egalitarian realization of global health goals, engage students as active participants in metacognition, and lead to an attitude shift invigorating students’ behavioral, emotional, cognitive, and agentic engagement (50).

Promoting students’ involvement made learning in this course real, meaningful, and enjoyable both for the teacher and the taught (51). This intuitive feeling resonated with another longitudinal study in the context of Irish curriculum reform that described the process of negotiation between teachers and students to promote learners’ agency around the decision-making of pedagogical activities (52).

The course importantly introduced the use of first-person trauma-informed storytelling as a continuum to contribute to the circle of care that, at one end, significantly impacts pedagogical effectiveness while also nurturing the best learning among students. It also facilitated to activate, transform, shift, trigger, and contradict critical insights that transformed a broader and deeper meaning-making of “classroom community” beyond merely a classroom space (53). Stories were not used as fixed or measurable information but as interpretable frames of meaning-making for teaching–learning that occurred through narrative co-constructions. Studies show that this is particularly important in the context of a NASNTI where students’ self-exploration and creating an engaging and exciting learning environment are deeply tied to their retention (54–56).

Photovoice, photo elicitation, and gratitude journaling made the instructor realize that these methods can set the stage for learners to share knowledge and experiences that can excite them about learning in a way that can make room for self-discovery, sensitize them to alternative voices, make them aware of individual, and can potentially create collective resilience in relation to a specific public health topic that can eventually foster connectedness between the students, professors, and external stakeholders (57, 58).

## Acknowledgment of constraints

We note several constraints in the storytelling pedagogic method, particularly during COVID-19. First, this pedagogic methodology was largely generative and “figured out” (59) during the COVID-19 pandemic, with several changes that occurred in critical college policies, teaching–learning modalities, and campus access procedures. The generalizability of practicing storytelling as a pedagogic strategy in institutions of higher education, especially in STEM courses, needs further affirmation. Delivering the course in a HyFlex format was seemingly useful for Native students who were unable to commute to school when COVID-19-induced curfews were in place in the reservation areas. This format also gave several quarantining students, those sharing one laptop for the whole household and those who needed to be in the reservation areas and take care of their families or work on the farm, the opportunity to listen to the Zoom recordings asynchronously. Digital storytelling as a strategic pedagogic approach was challenging during the COVID-19 pandemic for three main reasons. First, it required tremendous flexibility and dexterity of the instructor to separately design reflection and group exercises for in-class students and those using Zoom breakout rooms synchronously and those taking the class asynchronously for every single class. Second, the digital divide in this area impacted students’ class participation with some students from the Navajo Nation who had to drive miles to access the internet from a commercial parking lot and who had to climb to the top of hills near their homes to access Wi-Fi and others who were simply unable to access the internet. Last but not least, storytelling strategies relied heavily on synchronous, in-person, and consistent personal interaction between students, academics, and community members, to develop rapport. Thus, it was almost impossible to practice storytelling effectively during the peak of the COVID-19 pandemic (60).

## Conclusion

Summing up, using storytelling pedagogy in the Global Health course was in a way a contribution to the indigenous tradition of storytelling that provided an opportunity to ‘listen’ and ‘share’ perspectives and discover the layers of meaning-making of ‘reconciliation’ and ‘resilience’ among several marginalized communities across the world. Considering that such transformations are holistic, ongoing assessment and evaluation of expected outcomes are proposed to cultivate an interest in storytelling for teacher education candidates and students. Careful planning in the curriculum design measured, preferably through pre-post studies to evaluate personal and teaching benefits that arise from implementing storytelling in the classroom curriculum, and 360-degree assessments are proposed. More systematic reviews are recommended to identify and document gold standards and analyze interests, challenges, and applicability of storytelling pedagogic strategies in both humanities and natural and applied science courses. Capacity building of instructors and apportioned resources is recommended to develop and use story-based teaching tools in the class and assess students’ learning outcomes beyond the pandemic-induced application of this method (61, 62).

## Data availability statement

The original contributions presented in the study are included in the article/supplementary material, further inquiries can be directed to the corresponding author.

## Author contributions

TD: conceptualization, formal analysis, project administration, writing – original draft, review, editing, and finalization. CK and TD: literature review, investigation, methodology and reflections, and writing – review and editing. All authors contributed to the article and approved the submitted version.
